# The Academic Advantage: Gender Disparities in Patenting

**DOI:** 10.1371/journal.pone.0128000

**Published:** 2015-05-27

**Authors:** Cassidy R. Sugimoto, Chaoqun Ni, Jevin D. West, Vincent Larivière

**Affiliations:** 1 School of Informatics and Computing, Indiana University Bloomington, Bloomington, Indiana, United States of America; 2 School of Library and Information Science, Simmons College, Boston, Massachusetts, United States of America; 3 University of Washington Information School, Seattle, Washington, United States of America; 4 École de bibliothéconomie et des sciences de l’information, Université de Montréal, Pavillon Lionel-Groulx, Succ. Centre-ville, Montréal, Quebec, Canada; 5 Observatoire des Sciences et des Technologies (OST), Centre Interuniversitaire de Recherche sur la Science et la Technologie (CIRST), Université du Québec à Montréal, Succ. Centre-Ville, Montréal, Quebec, Canada; Universidad Veracruzana, MEXICO

## Abstract

We analyzed gender disparities in patenting by country, technological area, and type of assignee using the 4.6 million utility patents issued between 1976 and 2013 by the United States Patent and Trade Office (USPTO). Our analyses of fractionalized inventorships demonstrate that women’s rate of patenting has increased from 2.7% of total patenting activity to 10.8% over the nearly 40-year period. Our results show that, in every technological area, female patenting is proportionally more likely to occur in academic institutions than in corporate or government environments. However, women’s patents have a lower technological impact than that of men, and that gap is wider in the case of academic patents. We also provide evidence that patents to which women—and in particular academic women—contributed are associated with a higher number of International Patent Classification (IPC) codes and co-inventors than men. The policy implications of these disparities and academic setting advantages are discussed.

## Introduction

Innovation is critical to economic development [1] and depends upon the full participation of the scientific workforce [2]. Yet, the growing field of “innovation studies” [3] demonstrates that there are many disparities in the exploitation of human capacity for innovation. Two particularly well-noted areas are the dearth of academic and female innovators [4, 5]. The response to this lack of innovation in the academic sector has been to stress academic entrepreneurship, which encompasses the varied ways in which faculty at educational institutions engage in innovative and high risk activities which have the potential for financial rewards for the individual or the institution with which they are affiliated [6]. This is most typically operationalized as commercialization of science activities such as patenting [2], which was heavily promoted following the enactment of the Bayh-Dole Act in 1980 in the United States and similar initiatives in other countries [5].

Historical studies have shown that the rate of female patenting from 1637 to the mid-20^th^ century failed to exceed 2% of total patenting [7]. Contemporary studies suggest that women may continue to be underrepresented [4, 8, 9]; however, studies on rates of female patenting are largely monodisciplinary, localized, and lack explicit connections to the types of settings where the patenting is conducted. This study addresses this gap by providing a comprehensive analysis of 4.6 million utility patents issued between 1976 and 2013 by the United States Patent and Trade Office (USPTO). The data includes 10.8 million inventors and 4.2 million assignees (owners of the property of the patents that are different from inventors).

The need to understand diversity in patenting was stressed in the 2010 America Invents Act (AIA2010), which mandated the Director of the USPTO “establish methods for studying the diversity of patent applicants, including those applicants who are minorities, women, or veterans” [10]. The 2013 Federal Register [11] disclosed the first analysis of the 2005–2006 USPTO data, discussed the poor matching of inventors with Census data, and called for public comment on how to study the diversity of patent applicants pursuant to AIA2010. This paper answers the USPTO call by providing a comprehensive analysis of women in all USPTO patents, taking into account diversity by patent area and inventor country, and type of assignees.

## Materials and Methods

### Data Overview

This study imported the 4.6 million utility patents granted by the USPTO between 1976 and 2013 into an SQL database for analysis. Inventors from more than 185 countries invented patents issued by the USPTO. While most patents (53.44%) were invented by US inventors, 19.36% were from Japan, 7.08% from Germany, 2.6% from the UK, 2.43% from France, 2.22% from South Korea, 2.08% from Taiwan and 2.06% from Canada. On the whole, these 8 countries account for 91.37% of all inventorships of the dataset.

### Gender Identification

The identification of inventor gender is an essential task for this research. In this study, the gender classification of inventors in the USPTO database is perceived using the approach introduced in our earlier work [12]. The master list of name-gender assignments were created using universal and country-specific name lists. Universal lists were applied to the entire set of Web of Science authors, and country-specific lists were applied to subsets of Web of Science authors associated with the corresponding countries. This approach for name-gender classification has been manually validated [12]. For more detailed information on name-gender classification, please refer to [12].

**Fig 1:**
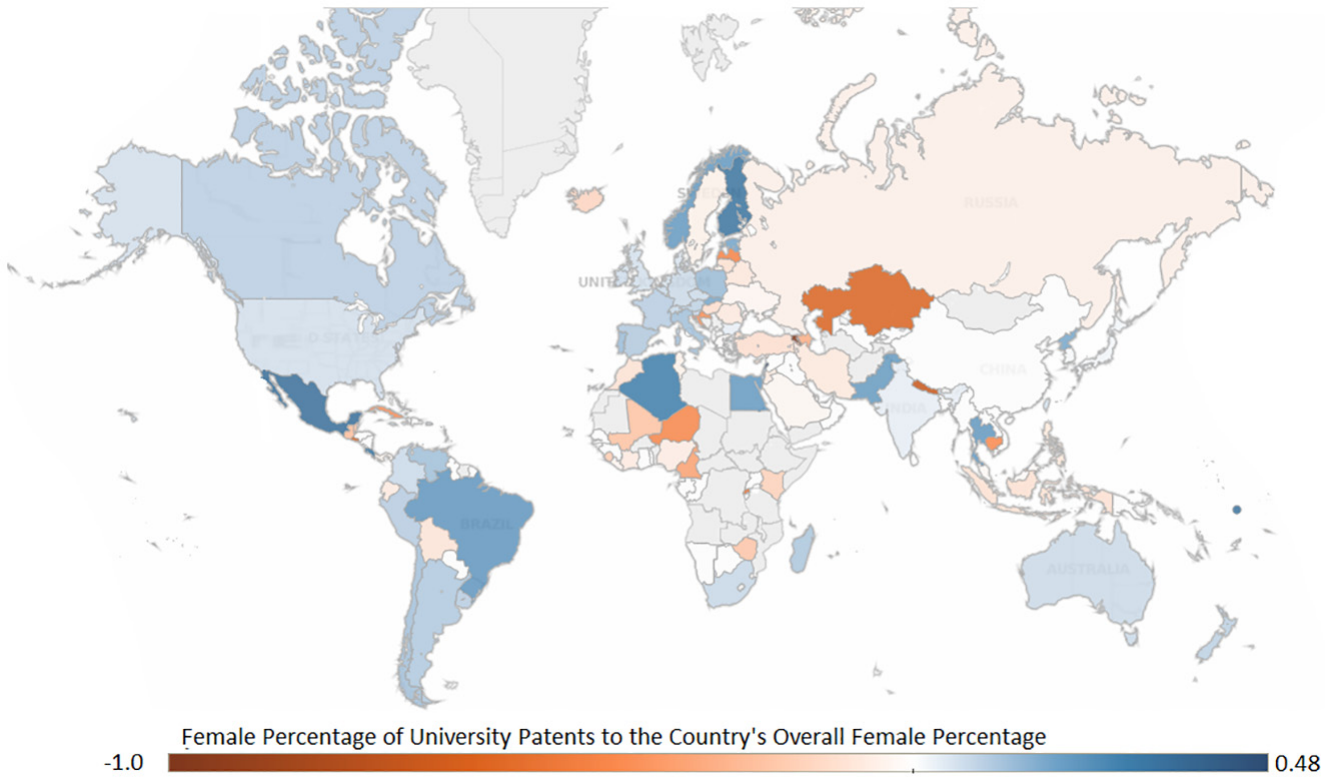
Proportion of inventor-patent combinations, by gender of inventor, 1976–2013.

The accuracy of the gender assignation table varies by country. For the United States for instance, 90.8% of inventor-patent combinations—that is the sum of all inventors’ names appearing on all patents—were assigned to a gender, leaving less than 10% of unassigned combinations, due to unisex names (2.0%), the use of initials only (0.1%) or to unknown names (7.1%). In addition to the United States, Germany, the United Kingdom, Italy and Australia have very high gender assignation rates, with more than 90% of all inventor-patent combinations assigned to either female or male. The assignation rate is much lower for India (66.1%), South Korea (66.4%), Taiwan (72.9%), and the Netherlands (72.9%). At the world level, 86.9% of all inventor-patent combinations were assigned to a gender. This proportion of assigned inventor-patent combinations has increased slightly over the period, from 10.8% to 15.0%, as a consequence of the internationalization of USPTO patenting (Fig 1).

### Classification of assignee types

Each entity was also placed into a type category: firm, academic, government, individual, and other. Those that could not be placed into one of these classifications were called “unclassified.” Each entity was manually examined and classed. However, to expedite and standardize the process, certain filters were used to locate entities for probable classification. For example, entities containing the term “gmbh” were likely corporate entities; similarly, those mentioning “university” were likely associated with an institution of higher education or those mentioning “represented”—as in “The United States of America as represented by the Administrator of the National Aeronautics and Space Administration”—were associated with the government sector. Individual patents contained names with the presence of semi-colons. The list of these filters is provided in Table 1 and was used to select items for manual identification. Those that could not be classified using these filters were manually searched online. In most cases, the first or second Google hit was either the agency's website or a Wikipedia entry, allowing the institution’s type to be easily determined. In some cases, an item could arguably be classed in more than one category (e.g., a veteran’s medical center could serve simultaneously as a government agency, a teaching institution, and a hospital). Therefore, we prioritized in the following way: if an entity conferred degrees or were associated with degree conferring institutions, it was classified as an academic institution. If it was not academic, we coded in the following order: 1) corporate, 2) hospital, 3) government, 4) nonprofit. There were very few institutions coded as “non-profit,” as the higher-priority categories accounted for most of the institutions represented in the list. In total, 89.2% of the 4,584,457 patents were classified into an institutional type. The type, number of entities associated with this type, the number of patents associated with these entities, and the percentage of all patents is listed in Table 2.

**Table 1:** Filters for institution type.

Firm	University	Government
& Cie or “et cie”	academy	Contains “represented”, as in “The Government Of The United States Of America As Represented By The Secretary, Department Of Health & Human Services, Center For Disease Control.”
(s.) AND (a.) [includes S.p.a]	college	government
“ag”	ecole	“secretary”
“lp”	polit/polyt	“united states”
“Oy”	“regents”	
“m.b” OR “m. b”	school	
a. AND g.	univ	
a/s OR a /s		
ab		
Aktiebolag		
Aktiengesellschaft		
Begins with “ab”		
bv		
bvba		
co.		
company		
Contains “products” AND DOES NOT contain “institute”		
Contains s.a.		
corp.		
corporation		
Ends with “ab”		
Ends with “as” or “sa”		
Ends with “* kg”		
Ends with “spa”		
gmbh		
inc		
“industries”		
k. [for k.k.]		
Kabushiki		
Kaisha		
l. AND p.		
limited		
llc		
llp		
ltd		
mbh		
nv		
plc		
s.r.l.		
Societe Anonyme		

**Table 2:** Number and percentage of assignee-patent combinations, by type.

Type	Patents	Percentage of total
Firm	3,470,845	72.4%
University	105,704	2.2%
Government	68,085	1.4%
Others	20,426	0.4%
Individual	612,405	12.8%
Unclassified	518,271	10.8%

### Technological classification of patents

Each patent issued by the USPTO is assigned to one or many technological classification codes based on the international patent classification (IPC, see: http://www.wipo.int/classifications/ipc/en) created by the World Intellectual Property Office (WIPO). The IPC is a 5 level hierarchical classification, which makes it very precise from a classification point of view, but much less relevant for data aggregation. In order to provide insight on the relationship between gender and technological technological classes, we applied the IPC8-Technology Concordance conversion table (updated January 2013), which groups technological innovation into 5 broad fields and 35 subfields, to the main IPC of each patent (http://www.wipo.int/ipstats/en/statistics/patents/pdf/wipo_ipc_technology.pdf).

### Fractionalized counting of inventorship by gender

This study utilized a fractionalized counting of inventorships by gender. That is, each inventor is given 1/*x* count of the inventorships where *x* represents the number of inventors for which a gender could be assigned on the given patent. This method tries to eliminate possible inflations brought by patents with a large number of inventors. This fractionalized counting of inventorships by gender are then aggregated at the country level to denote the patent performance by gender in each country, at sector level to illustrate the patent productivity by gender by sector, and at the technological area level to denote the patent productivity by gender by technological area.

### Patent field-normalized impact

The citations received each patent is considered as an indication of that patent’s impact. To enable the comparison of patent impact across areas, the number of citations each patent received is normalized by field, as has been described in [12]. That is, each patent’s number of citation is divided by the average number of citations received by patents in the same technological areas—using the IPC concordance table—issued that year. When the average of relative citations (ARC) is above 1, a given patent is cited above the world average for the same field. Conversely, an ARC below 1 means that the number of citations received is below the world average.

## Results

**Fig 2:**
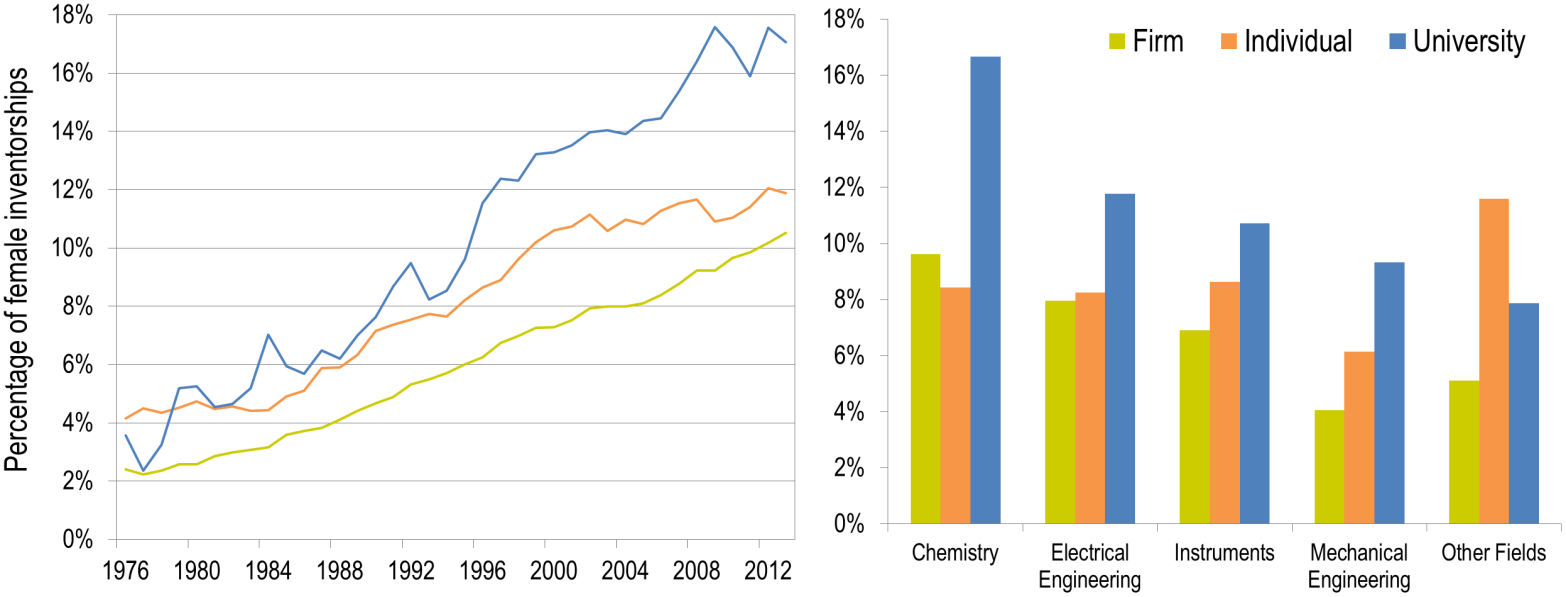
Ratio between the proportion of women inventorships of patents owned by the university sector and the proportion of the women inventorships overall in that country.

Women contributed less than 8% of all inventorships for the entire period (1976–2013) and contributed 10.8% in the most recent year (2013); an increase from 2.7% in 1976. Male dominance in patenting is found in nearly every country, with 42 countries listing no female inventors (Fig 2). Only five countries are female dominated, and these countries all have fewer than 35 fractionalized patents.

### Setting

**Fig 3:**
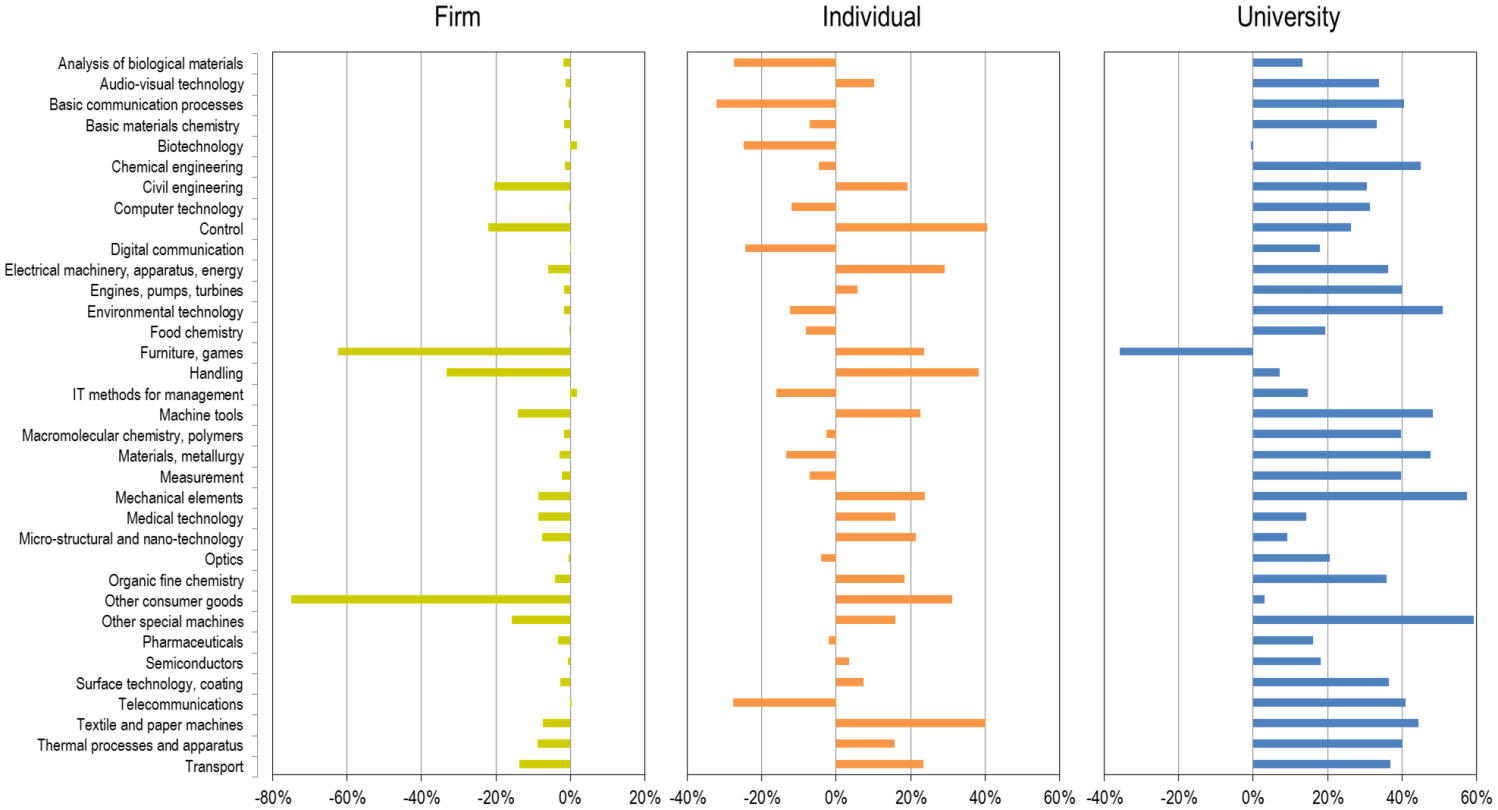
Proportion of female inventorships, by type of assignee and year (left panel) and by macro technological area (right panel), 1976–2013.

Women are the minority in every technological area. However, significant differences can be seen by setting (i.e., firm, individual, university), with patents owned by universities demonstrating the highest mean female participation (at 11%, compared to less than 8% in firms). Fig 3 presents the proportion of female inventorship over time, by setting, and by macro-disciplinary area. As shown (Fig 3, left panel), the difference across settings has been increasing over time: while in 1976, females accounted for about 2–3% of both industry and university’s patents, the difference between the two sectors became more apparent in the early 1990s. By 2013, women inventorships accounted for 18% of university-owned patents, compared to 10% for industries and 12% for individuals. Higher proportions of academic female inventorships are apparent across all macro technological areas, with the exception of “Other fields”, where patents owned by individuals have a higher share of female inventorships, mainly due to technological areas such as furniture, games, and other consumer goods (Fig 3, right panel).

### Specialties

**Fig 4:**
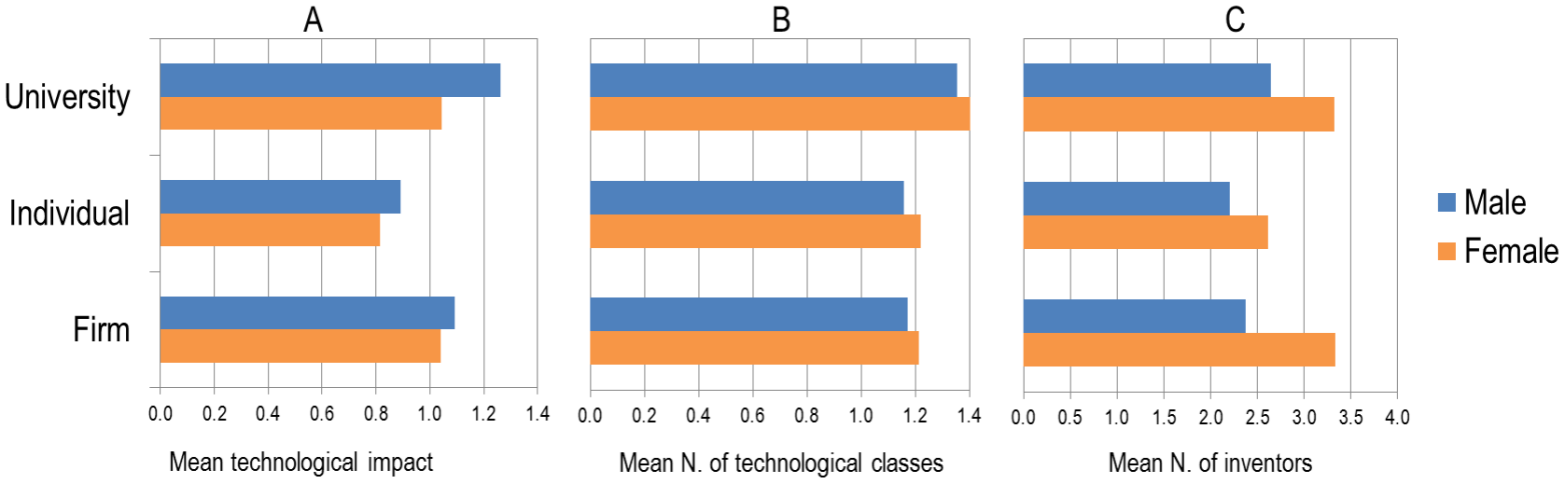
Difference from the world average of female inventorship, by technological area and type of assignee, 1976–2013.

It might be suggested that the higher proportion of female patenting in universities is actually the effect of an emphasis on certain technological areas which have a disproportionate number of females (e.g., biotechnology). To address this, Fig 4 displays, for each of the types of assignees and technological area, the difference between their percentage of female inventorships and the female inventorship of the technological area at the world level. Therefore, for a specific type of assignee and technological area, a value below zero means its percentage of female inventorship is below the world average, and a value above zero means the opposite. As shown, the strength of female patenting in academic settings persists across almost all technological areas, whereas firms are consistently below the world average in terms of female participation by specialty.

### Impact

**Fig 5:**
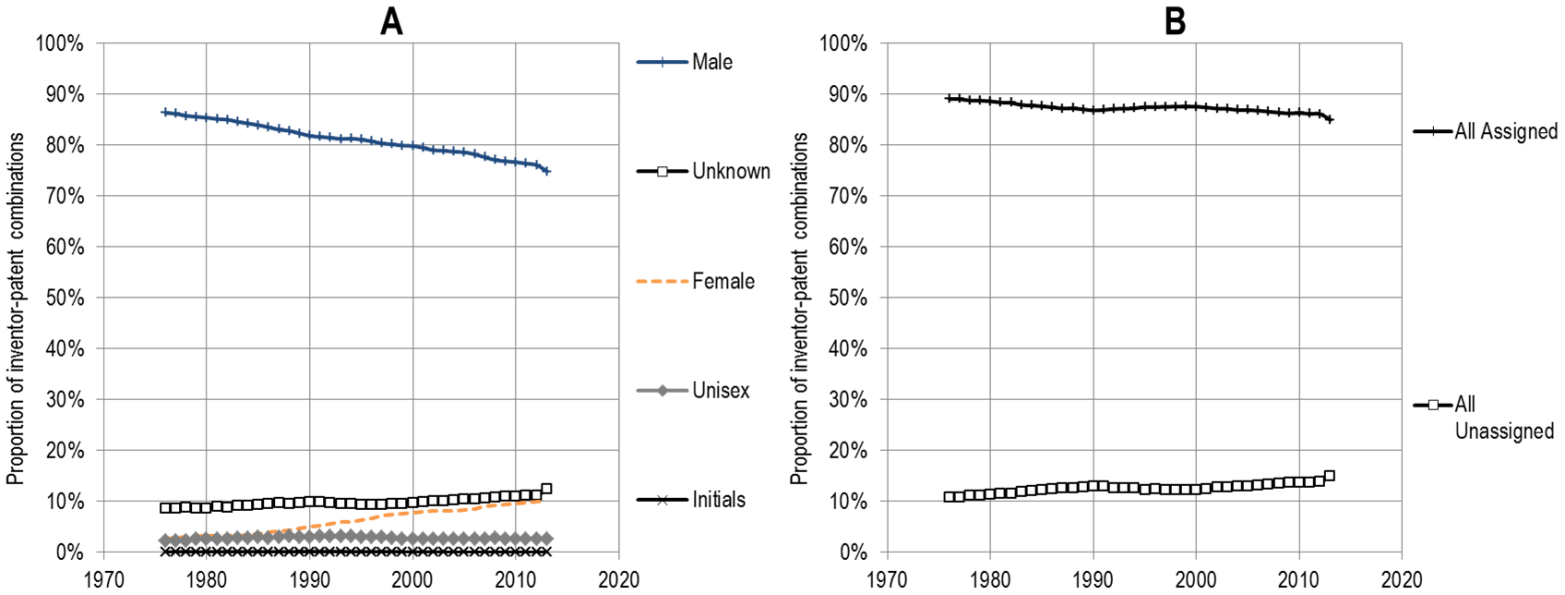
A) Mean field-normalized technological impact (i.e., citations), B) Mean number of technological classes and C) Mean number of inventors, of patents to which female and male contributed, by type of assignee, 2000–2013.

The gender gap in terms of scientific impact has been well-documented [12]. However, little is known about the technological impact gap, i.e., the number of times women’s patents are cited in other patents compared to that of men. Fig 5A presents the mean number of citations per patent (i.e., mean technological impact): as shown, such impact is always lower for women’s patents, irrespective of the type of assignee. However, this gap is narrower for patents owned by firms, and larger for patents owned by the university sector, which suggests that the narrow gap in female participation in academic patents does not translate into a smaller technological impact gap. Patents by females obtain similar impact when owned by firms or by universities; the increased gender difference is due to male’s university-owned patents obtaining a higher impact than those owned by industries or by individuals. This might be explained by the well documented propensity of male academics to self-cite [13] and the fact that academic males represent the most frequent contributors to published research.

### Interdisciplinarity

It has been suggested that, when female inventors are involved, patents tend to have higher diversity in terms of the number of International Patent Classification (IPC) codes assigned [14]. This holds true in our data (Fig 5B): female inventors were associated with more IPC codes, irrespective of the type of assignee, suggesting higher interdisciplinarity in female patenting. This difference remains significant even when controlling for the concomitant increase in IPC codes and women over time and is found for all the top 10 most productive countries (in terms of number of patents), with the exception of Taiwan. In addition, female inventors have more co-inventors on average than male inventors in all IPC classes and this is observed for every type of assignee (Fig 5C). This heightened collaboration may be a behavioral difference (i.e., higher propensity to work in groups among women) or a social difference (i.e., women are only likely to patent when men are involved).

## Conclusions

These results demonstrate that women’s patenting remains lower than would be predicted given their representation in science, technology, engineering, and mathematics fields and professions and their authorship of scientific papers (where they represent about 33% of researchers [15] and 30% of authorships [12]). Furthermore, our study suggests that academic environments may be more conducive for female patenting than corporate or government organizations. This may be due to differing numbers of women in the workforce in each of these setting [15]. The higher degree of female patenting in academe may also be due to the less hierarchical organization of academic institutions [16], which have been shown necessary for building social networks—critical in fostering commercialization activity [17].

The lack of a strong social network has been repeatedly cited as a main reason for the suppressed commercialization activities of women [6, 18, 19, 2]. One way in which universities have responded to this is the creation of Technology Transfer Offices (TTOs), which were established to meet the demands of the Bayh-Dole Act in promoting the commercial exploitation of inventions that result from government-funded research [6]. Since the Bayh-Dole Act, there has been a tenfold increase in the number of TTOs at universities [20]; this might explain the increase in the proportion of women inventorships over the last decades. Strong TTOs have been suggested as another approach to fostering academic entrepreneurship through organizational support and facilitating the construction of collegial networks [4]. They may also lead to reduced perceptions of risk, which has been shown to have gendered dimensions [21]. However, the degree to which TTOs are providing advantages for underrepresented innovators and taking into account the differential needs of some of these populations is yet unknown [22]. Another potential reason for the higher proportion of female patents in universities is the lower probability of university patents to be defensive or associated with ‘patent trolls’, although this might change in the future [23].

Patenting, of course, does not encompass the entire spectrum of innovative activities. However, women seem to be at a disadvantage across this spectrum: while they might be included on publications related to the patent, women’s names disappear between the article about the patent and the patent itself [24] and even fewer women see the commercialization and licensing of their patents [2].There remains much to be done in terms of active and equitable academic entrepreneurship. Universities have slowly begun including “inventions” as plausible products to include in tenure and promotion portfolios [25]. However, patenting is still seen as an optional activity, which may cause women (particularly those with children) to “opt out” [26]. Given that patenting is not at the expense of publishing, but rather that the two are positively correlated [27, 28, 2], it would benefit universities to promote patenting among all its scholars, particularly as universities are under increased scrutiny from the public and policy makers to produce goods and engage in activities with demonstrable economic impact [6].

One may question whether patenting is an inherently positive thing and should be promoted. However, regardless of whether it is desirable, it has become an established component of contemporary science and another metric where inequality can affect subsequent accolades in the academy. As long as institutions are promoting and rewarding this activity, it’s critical that potential disparities in the system are recognized and addressed.
